# Bioinspired Fern-like Fe_2_O_3_ Functionalized with Pd/PdO Nanoparticles for High-Performance Acetone Sensing

**DOI:** 10.3390/molecules29235791

**Published:** 2024-12-07

**Authors:** Gaohan Liu, Haihang Wang

**Affiliations:** 1 College of Materials Science and Engineering, Qingdao University, Qingdao 266071, China; xiaonuanhan@163.com; 2 College of Materials Science and Engineering, Liaocheng University, Liaocheng 252000, China

**Keywords:** fern-like sensor structure, acetone sensing, metal oxide gas sensors, functionalized Pd/PdO-Fe_2_O_3_ gas sensors

## Abstract

The accurate monitoring and detection of acetone vapor are essential for environmental and human safety. Consequently, fern-like Fe_2_O_3_ with hierarchical vein-like structures is synthesized via a concise hydrothermal method. Compared with pure fern-like Fe_2_O_3_, fern-like Pd/PdO-Fe_2_O_3_ shows the best acetone-sensing characteristics, in terms of lower operating temperature (180 °C), better selectivity and excellent long-term stability. More importantly, the response value of the Pd/PdO-Fe_2_O_3_ sensor to 100 ppm acetone reaches as high as 73, which is 55% higher than that of pristine fern-like Fe_2_O_3_. This enhanced sensing performance can be ascribed to the synergistic effect between Pd/PdO and fern-like Fe_2_O_3_. On the one hand, Pd/PdO nanoparticles show favorable catalytic activity toward ionized oxygen molecules; meanwhile, the formation of the heterojunction between PdO and fern-like Fe_2_O_3_ plays an important role. On the other hand, the hierarchical nature of fern-like Fe_2_O_3_ promotes efficient gas diffusion throughout the structure. Based on its advantages, fern-like Pd/PdO-Fe_2_O_3_ becomes a satisfactory candidate for acetone gas sensors.

## 1. Introduction

Acetone is a colorless, volatile organic compound that poses significant health and environmental risks. It is widely used in industrial processes and products such as solvent production, paint thinners, and chemical synthesis, leading to its release into the atmosphere [1,2]. Prolonged exposure to acetone can cause various adverse health effects, including headaches, dizziness, respiratory issues, and skin irritation [3,4,5]. Furthermore, acetone is also a critical biomarker in medical diagnostics, particularly for diabetes management. Elevated levels of acetone in breath can indicate ketosis, a condition associated with uncontrolled diabetes [5,6]. Therefore, the accurate monitoring and detection of acetone are essential not only for environmental safety but also for early diagnosis and the management of health conditions [7,8,9]. Given the potential hazards associated with acetone, there is a pressing need to develop effective acetone sensors. Traditional detection methods, such as gas chromatography, are often expensive and require sophisticated laboratory setups, making them impractical for real-time monitoring [10,11,12]. As a result, the development of reliable, cost-effective, and portable acetone sensors has become a key focus in research and industrial applications.

Metal oxides (MOs) have emerged as promising materials for gas-sensing applications, particularly due to their sensitivity, stability, and ease of fabrication [13,14,15,16]. Among the various metal oxide sensors (MOSs), iron oxide (Fe_2_O_3_) has gained attention as a suitable candidate for acetone detection due to its favorable electronic properties, abundance, and low cost [17,18,19]. However, while Fe_2_O_3_ demonstrates promising sensing capabilities, its performance can be limited by factors such as poor selectivity, high operating temperatures, and inadequate response times [20,21,22]. Therefore, optimizing its morphology and enhancing its catalytic properties are crucial for improving acetone-sensing performance.

One effective strategy for optimizing the sensing performance of metal oxides is through morphological control. By manipulating the nanoscale structure of metal oxides, researchers can significantly enhance gas diffusion and surface interaction with acetone molecules [23,24,25,26]. For instance, hierarchical Fe_2_O_3_ structures with various morphological properties provide enhanced gas diffusion pathways [27,28]. Similarly, porous NiO architectures offer abundant oxygen adsorption sites, promoting stronger interactions with acetone [29].

The incorporation of noble metals such as silver (Ag) [30,31], platinum (Pt) [32,33,34], and palladium (Pd) [12,35,36] into these semiconductor matrices can significantly enhance their catalytic properties. These noble metals are known for their superior catalytic activity, which can facilitate reactions at lower temperatures [37,38,39,40,41]. For instance, silver has shown excellent performance in various oxidation reactions, while platinum and palladium are particularly effective in promoting electron transfer processes [37,38,42,43,44]. The formation of heterojunctions between these noble metals and semiconductor materials can create efficient charge separation, further enhancing overall catalytic efficiency and sensor performance [45,46,47].

In this work, by optimizing the morphological characteristics of Fe_2_O_3_ and integrating Pd/PdO to exploit their combined catalytic properties and heterojunction structure, we design and fabricate innovative sensing materials that meet the growing demand for effective and reliable acetone detection. This research not only contributes to the advancement of gas-sensing technology but also highlights the importance of interdisciplinary approaches in addressing real-world challenges.

## 2. Experimental Procedure

### 2.1. Preparation of Fern-like Fe_2_O_3_ and Fern-like Pd/PdO-Fe_2_O_3_

**Figure 1:**
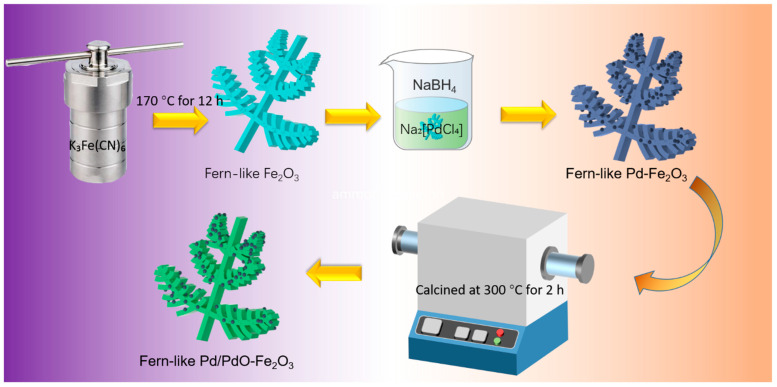
Schematic illustration of the preparation process of Fern-like Pd/PdO-Fe_2_O_3_.

The preparation process is displayed in Figure 1. Specifically, 0.5 g of K_3_Fe(CN)_6_ was dispersed in 70 mL of distilled water to create a suspension, and the pH was adjusted to 8.0 using 0.1 M ammonia solution. This mixture was then transferred to a 100 mL Teflon-lined stainless-steel autoclave, which was sealed and heated at 170 °C for 12 h before cooling naturally. The resulting product was collected via centrifugation, washed multiple times with deionized water and absolute ethanol, and finally dried in a vacuum at 60 °C, yielding red fern-like Fe_2_O_3_. As for fern-like Pd/PdO-Fe_2_O_3_, 0.05 g of fern-like Fe_2_O_3_ microspheres was dispersed in deionized water under ultrasonic treatment, followed by the addition of 1 mL of Na_2_[PdCl_4_] (2 mmol L^−1^) with vigorous agitation. Then, 1 mL of deionized water containing 0.4 mg of NaBH_4_ was added dropwise to the mixture. After being stirred for approximately 2 h, the mixture was washed with deionized water and absolute ethanol, and then centrifuged. The final powder was calcined at 300 °C for 2 h and labeled as fern-like Pd/PdO-Fe_2_O_3_.

### 2.2. Characterization

The characterizations of fern-like Fe_2_O_3_-based samples were performed using several advanced techniques. X-ray diffraction (XRD) was conducted with a Bruker D8 Advance (Billerica, MA, USA) to analyze crystalline structure, while X-ray photoelectron spectroscopy (XPS) using a Thermo Scientific K-Alpha (Waltham, MA, USA) was employed to determine the surface chemical states and elemental composition. Morphology was examined using scanning electron microscopy (SEM) (JEOL JSM-7500F, Akishima, Japan) and complemented with energy-dispersive spectroscopy (EDS) mapping for elemental distribution. Transmission electron microscopy (TEM) (JEOL JEM-2100F) provided insights into internal structures and particle size, with high-resolution transmission electron microscopy (HRTEM) on the same instrument delivering detailed information on lattice spacing and crystallinity. Together, these techniques offered comprehensive insights into the structural and compositional features of the synthesized material, highlighting its potential applications in gas sensing and catalysis.

### 2.3. Gas Sensor Fabrication and Gas-Sensing Properties Test

The sensor fabrication process involved mixing Fe_2_O_3_-based sensing materials with ethanol in a 4:1 weight ratio and then grinding the mixture for 20 min to form a paste. This paste was then evenly applied to the surface of a tube with gold electrodes to create the sensing layer. After coating, a Ni-Cr alloy coil was inserted into the ceramic tube to serve as a heater, ensuring precise temperature control. The process concluded with the connecting of the necessary junctions to the sensor sockets. Gas-sensing properties were tested with the WS-60A gas-sensing characterization system (Weisheng Instruments Co. Ltd., Shenzhen, China) using the static liquid–gas distribution method for various gas concentrations, as detailed in the Supplementary Materials. The sensor response (R) is defined as R = R_a_/R_g_, where R_g_ and R_a_ represent sensor resistance in the target gas and in dry air, respectively. Response time and recovery time are defined as the time taken to reach 90% of the total resistance change during adsorption and desorption, respectively. To confirm the thickness of the sensing layer, a diagram of its cross-section is illustrated in Figure S1. The thickness of Pd/PdO-Fe_2_O_3_ is about 20 μm.

## 3. Results and Discussion

### 3.1. Structural and Morphological Properties

**Figure 2:**
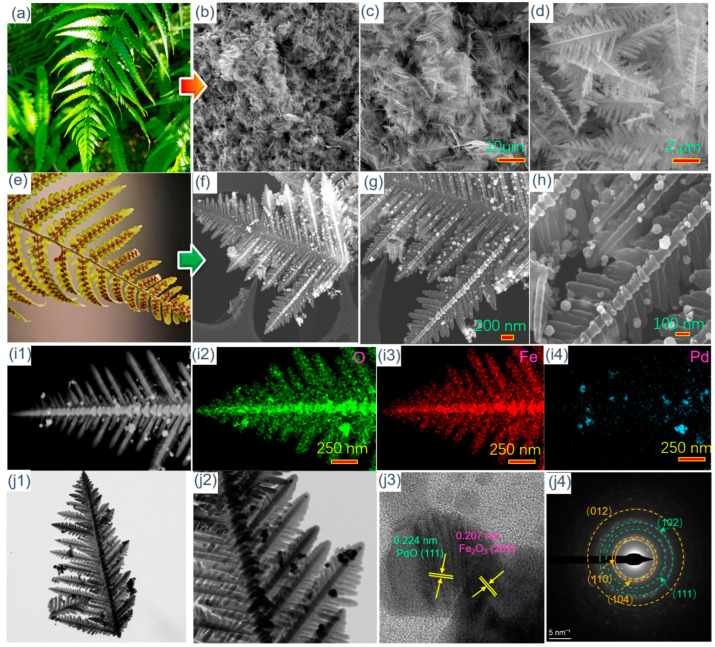
(**a**) An optical figure of the fern blade in (**a**) spring and (**e**) autumn; SEM images of (**b**–**d**) fern-like Fe_2_O_3_ and (**f**–**h**) fern-like Pd/PdO-Fe_2_O_3_; (**i1**) HAADF image and the corresponding EDS elemental mapping images of (**i2**) O, (**i3**) Fe, and (**i4**) Pd, respectively; (**j1**–**j2**) TEM, (**j3**) HRTEM images, and (**j4**) area diffraction pattern of fern-like Pd/PdO-Fe_2_O_3_.

Figure 2a shows an optical figure of the fern blade in spring. Based on its vein structure, fern-like Fe_2_O_3_ is designed and synthesized using a concise hydrothermal method. The morphology and microstructure of fern-like Fe_2_O_3_ are investigated by SEM analysis and the results are illustrated in Figure 2b–d. It can be seen from Figure 2b,c that there are many uniform and independent fern-like Fe_2_O_3_ structures. A fern-like structure consists of a stem with two groups of highly symmetric, parallel branches. Moreover, the angle between the veins and leaves is about 55°, which is extremely similar to that of the fern blade. This result confirms the successful preparation of fern-like Fe_2_O_3_. Differently from the green fern blade, the seeds of the fern will grow evenly on the leaves again in the autumn, which allows it to obtain a larger contact area so that the seeds are spread efficiently (Figure 2e). In order to achieve higher gas-sensing efficiency, the Pd/PdO nanoparticles are decorated on the surface of fern-like Fe_2_O_3_. The surface properties of Pd/PdO-Fe_2_O_3_ are presented in Figure 2f–h. After the reduction and calcination processes, the Pd/PdO-Fe_2_O_3_ retains the initial morphology of fern-like Fe_2_O_3_. Specifically, as shown in Figure 2h, the Pd/PdO nanoparticles with diameters of 20–50 nm are evenly distributed on the fern-like Fe_2_O_3_ surface. In addition, Pd/PdO-modified fern-like Fe_2_O_3_ is characterized using element mapping images. The presence of Fe, O, and Pd can be clearly observed in Figure 2(i1–i4). The distribution of Fe and O is fully consistent with the fern-like hierarchical structure. Meanwhile, the Pd element tends to be evenly dispersed on the fern-like Fe_2_O_3_ leaf. The microstructure of fern-like Pd/PdO-Fe_2_O_3_ is explored by TEM and HRTEM, and the images are shown in Figure 2(j1–j4). The light region corresponds to the fern-like Fe_2_O_3_, while the dark dots represent the Pd/PdO nanoparticles. As shown in Figure 2(j1–j2), all the Pd/PdO nanoparticles are uniformly decorated on the Fe_2_O_3_ surface, without the existence of serious agglomeration behavior, which can occur upon direct contact with the target gas molecules. To achieve more detailed visualization of Pd/PdO-modified fern-like Fe_2_O_3_, HRTEM is conducted to explore the interface characteristics of fern-like Pd/PdO-Fe_2_O_3_, as demonstrated in Figure 2(j3). Obviously, it is easy to observe the interface between the Pd/PdO nanoparticles and fern-like Fe_2_O_3_, in which the fringe spacing values of 0.224 nm and 0.207 nm can be assigned to the PdO (111) and Fe_2_O_3_ (202) planes, separately. Figure 2(j4) illustrates the selected area diffraction pattern, and clear diffraction is presented in this figure. These diffraction rings correspond to the (110), (104), and (012) lattice planes of the Fe_2_O_3_ and the (111) and (102) lattice planes of the Pd/PdO cubic structure. This verifies the polycrystalline nature of the nanoparticles Pd/PdO and fern-like Fe_2_O_3_.

**Figure 3:**
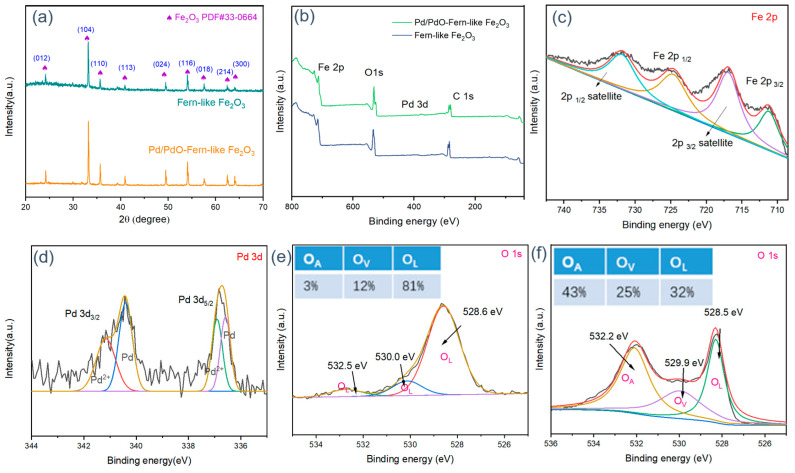
(**a**) XRD pattern and (**b**) XPS spectrum of Fe_2_O_3_ and Pd/PdO-Fe_2_O_3_; high-resolution XPS spectrum of (**c**) Fe 2p, (**d**) Pd 3d of Pd/PdO-Fe_2_O_3_; O 1s spectrum of (**e**) Fe_2_O_3_ and (**f**) Pd/PdO-Fe_2_O_3_.

Figure 3a reveals the XRD pattern of the original fern-like Fe_2_O_3_ and fern-like Pd/PdO-Fe_2_O_3_ prepared using the hydrothermal and reduction methods. It can be seen that the diffraction peaks of all samples match well with the rhombohedral structure of α-Fe_2_O_3_ (JCPDS PDF# 33-0664). The strong characterization peaks located at two-theta angles of 24.1°, 33.2°, 35.6°, 40.9°, 49.5°, 54.1°, 62.5°, 68.3°, 70.1°, and 74.0° can be indexed to the (012), (104), (110), (113), (024), (116), (214), (300), (220), and (018) crystal planes of α-Fe_2_O_3_, respectively. In addition, the intensity of peaks for α-Fe_2_O_3_ increases dramatically after high-temperature calcination. This change is often due to variations in crystallinity. Higher temperatures typically promote grain growth, leading to sharper and more intense peaks. However, there is no appearance of typical diffraction peaks for Pd/PdO, which is related to the low-loaded mass of Pd/PdO on the surface of α-Fe_2_O_3_. No other impurity peak can be observed across the entirety of the XRD pattern, demonstrating that the synthesized Fe_2_O_3_ sample is a typical composite consisting of Pd/PdO and Fe_2_O_3_ with excellent purity. The XPS analysis was conducted to explore the surface composition and valence state of all Fe_2_O_3_ products. As shown in Figure 3b, the full survey spectra of pristine Fe_2_O_3_ and Pd/PdO-Fe_2_O_3_ consist of the Fe, O, and Pd elements. Figure 3c shows the high-resolution Fe 2p spectrum, with bending energies at 710 eV and 725 eV corresponding with Fe 2p_3/2_ and Fe 2p_1/2_, which coincides well with the XRD analysis. Meanwhile, the bonding energy at 718 eV and 732 can be ascribed to the satellite peaks. As depicted in Figure 3d, two kinds of characterization peaks can be found, which attribute corresponding energies of 336.1 and 337.8 eV to the metal Pd and PdO, respectively. In fact, both Pd and PdO reveal excellent catalytic activity toward the acetone, which originates from the calcination treatment of metal Pd nanoparticles. To confirm the effect of decoration with Pd/PdO nanoparticles on Fe_2_O_3_, different contents of the various oxygen species are investigated comprehensively. The O 1s spectra can be divided into three typical peaks, which consist of lattice oxygen (O_L_), vacancy oxygen (O_V_), and adsorbed oxygen (O_A_), respectively. The percentages of the surface oxygen species are listed in Figure 3e,f. It is obvious that the contents of O_V_ and O_A_ increase from 12% to 25% and 3% to 43%, respectively, after surface optimization with Pd/PdO nanoparticles. The increased O_A_ content is beneficial in enhancing gas-sensing performance by offering additional ionized oxygen species. The large increase in surface-adsorbed oxygen species is due to the existence of the high catalytic activity of Pd/PdO nanoparticles.

### 3.2. Sensing Performance

**Figure 4:**
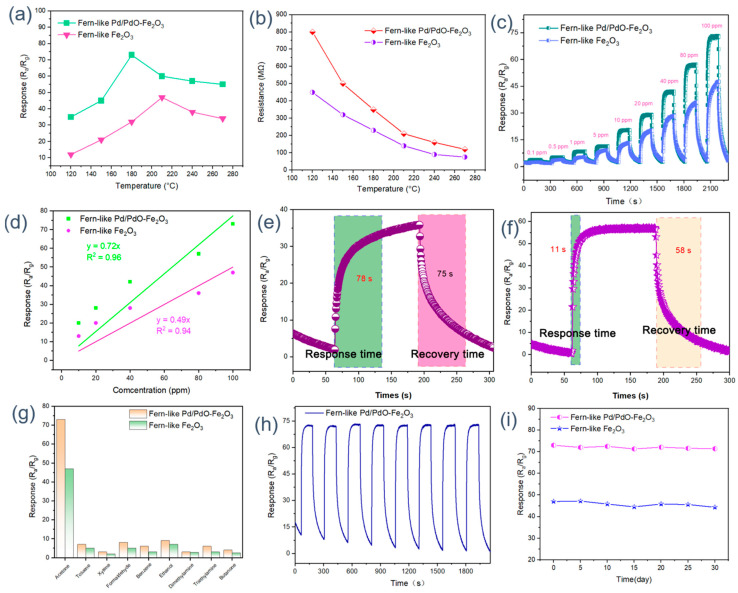
(**a**) Response and (**b**) resistance of all sensors to 100 ppm of acetone at different working temperatures; (**c**) dynamic gas response curves and (**d**) corresponding liner fitting curves of all sensors under different acetone concentrations; response–recovery time of (**e**) pure Fe_2_O_3_ and (**f**) Pd/PdO-Fe_2_O_3_ at 80 ppm acetone; (**g**) selectivity, (**h**) reproducibility, and (**i**) long-term stability of Fe_2_O_3_-based sensors.

In general, operating temperature has a significant influence on the gas-sensing performance of oxide semiconductors. Therefore, the temperature-dependent responses of Fe_2_O_3_ sensors toward 100 ppm acetone are recorded to confirm the optimum working temperature. As illustrated in Figure 4a, with increasing temperature, all the Fe_2_O_3_ sensors show the response tendency of ‘volcano’ and achieve a maximum response for Fe_2_O_3_ (47) and Pd/PdO-Fe_2_O_3_ (73) at 210 °C and 180 °C, respectively. As the sensor works at a low operating temperature, adsorbed gas molecules lack sufficient activity to trigger their gas-sensing reactions, leading to reduced reaction efficiency and lower sensor responses. As optimal operating temperature is reached, the adsorbed gas molecules can obtain enough energy to accelerate the gas-sensing reaction, resulting in a dramatic increase in the response values. Conversely, when the sensing device works in a high-temperature environment (beyond optimal temperature), the gas adsorption state would transform into desorption, leading to decayed sensing performance. Moreover, it can be seen that the optimum working temperature for Pd/PdO-Fe_2_O_3_ is lower than that for pure Fe_2_O_3_. This is due to the enhanced catalytic activity of Pd/PdO, which reduces the activation energy required for gas interactions. Additionally, the modification alters the surface properties of Fe_2_O_3_, increasing the availability of active sites for gas adsorption. As shown in Figure 4b, the initial resistance values in air of Fe_2_O_3_ and Pd/PdO-Fe_2_O_3_ are obtained at different working temperatures in the range from 120 to 270 °C. It is clearly observed that the original resistance values of all sensing devices decrease with increasing operating temperature. In addition, the initial resistance values of Pd/PdO-Fe_2_O_3_ are higher than those of the pristine Fe_2_O_3_ sensor. This can be attributed to the electronic properties introduced by Pd/PdO. Pd/PdO can indeed influence resistance by forming heterojunctions at the PdO/Fe_2_O_3_ interface [29,48]. These heterojunctions introduce energy barriers, which modify the charge carrier dynamics [49]. Additionally, the incorporation of these metallic phases can lead to changes in band structure and charge carrier mobility, resulting in enhanced electron scattering effects. Consequently, the increased resistance in Pd/PdO-Fe_2_O_3_ reflects the complex interactions between the semiconductor properties of Fe_2_O_3_ and the metallic characteristics of palladium. The dynamic response–recovery curves of all the sensors to different acetone concentrations (0.1 ppm–100 ppm) at the optimum operating temperature are displayed in Figure 4c. It can be seen that the Pd/PdO-Fe_2_O_3_ sensor shows the highest response toward acetone under the full concentration range. Figure 4d demonstrates the linear fitting curves of the sensitivity of the two sensors to the acetone concentration. The linear fitting curves for Fe_2_O_3_ and Pd/PdO-Fe_2_O_3_ are represented by the equations y = 0.33x + 11.8 (R^2^ = 0.98) and y = 0.55x + 16.5 (R^2^ = 0.99), respectively. Obviously, the response of the Pd/PdO-Fe_2_O_3_ sensor showed the highest fitting correlation coefficient in accurately detecting the concentration of acetone. When considering the practical application of gas sensors, the response–recovery efficiency is an important parameter to be investigated. Figure 4e,f display the response–recovery dynamic curves of Fe_2_O_3_ and Pd/PdO-Fe_2_O_3_ to 80 ppm acetone. The response–recovery times of the pristine Fe_2_O_3_ and Pd/PdO-Fe_2_O_3_ are 78/75 s and 11/58s, respectively. Compared to the initial Fe_2_O_3_ sensor, it can be observed that the incorporation of Pd/PdO nanoparticles dramatically shortens the response–recovery time. This high response–recovery efficiency can be ascribed to the excellent catalytic capability of the Pd/PdO nanoparticles, which can promote the dissociation of the oxygen molecules, therefore enhancing the surface gas-sensing reaction. The adsorbed oxygen species originating from the O1s XPS spectrum also confirms this inference. The selectivity measurements are conducted through the detection of 100 ppm of various gases, including acetone, toluene, xylene, formaldehyde, benzene, ethanol, and methanol, respectively. As revealed in Figure 4g, the Pd/PdO-Fe_2_O_3_ sensor shows the highest response to all detected gases, verifying the general increase in gas response with the introduction of Pd/PdO nanoparticles. Moreover, it is worth noting that the sensor based on the Pd/PdO-Fe_2_O_3_ composite has the highest response toward acetone among the various gas molecules, demonstrating good selectivity for acetone gas. In addition to their high gas-sensing properties, the conductometric gas sensors should also possess good reproducibility and long-time stability in actual application. Thereby, the repeatability and long-term stability of Fe_2_O_3_-based sensors are also explored by applying them to detect 100 ppm acetone at an optimum temperature. As depicted in Figure 4h, after eight successive response–recovery cycles, the Pd/PdO-Fe_2_O_3_ sensor response is still basically consistent with its first cycle value, indicating the good accuracy and reproducibility of the Pd/PdO-Fe_2_O_3_ sensor. Subsequently, the long-term stability of Fe_2_O_3_ and Pd/PdO-Fe_2_O_3_ sensing devices is investigated by comparing response values (the recording period is five days). The response values over 30 days remain basically unchanged, with the fluctuation being less than 3%, demonstrating that the Pd/PdO-Fe_2_O_3_ sensor has good long-term stability. In addition, a test of the humidity resistance capability of the Pd/PdO-Fe_2_O_3_ sensor is conducted as shown in Figure S2. Obviously, it can be seen that the sensor based on Pd/PdO-Fe_2_O_3_ shows excellent humidity resistance performance.

### 3.3. Sensing Mechanism

It is well known that the gas-sensing performance of a metal oxide semiconductor mainly depends on the resistance change resulting from the adsorption and desorption of gas molecules. Owing to the properties of n-type semiconductors, when the Fe_2_O_3_-based sensor is exposed to air, the adsorbed oxygen molecules capture electrons from the material conduction band and then ionize into oxygen species containing $O_{2}^{-}$, O^−^, and O^2−^ (Equations (1)–(3)). Meanwhile, an electron depletion layer is generated on the surface of the sensing materials, which decreases the electron concentration, increasing the resistance value. Because the working temperature of the Fe_2_O_3_-based sensor is below 300 °C, O^−^ is the dominant oxygen species. When the sensor fabricated by Fe_2_O_3_ is in contact with acetone molecules, the ionized oxygen molecules will interact with acetone molecules and return the free electrons to the sensing materials. This will lead to a decrease in the electron depletion layer and thus a decline in the resistance of the Fe_2_O_3_ sensor (Equation (4)).

(1) $$O_{2(\mathrm{ads})}+e^{-}\to O_{2}^{-}(T<100\;{}^{\circ}C)$$

O_2(ads)_ + 2e^−^ → 2O^−^_(ads)_ (100 °C < T < 300 °C) (2)

O_2(ads)_ + 4e^−^ → 2O^2−^ _(ads)_ (300 °C < T) (3)

CH_3_COCH_3_ (ads) + 8O^−^ (ads) → 3CO_2_ + 3H_2_O + 8e^−^ (4)

**Figure 5:**
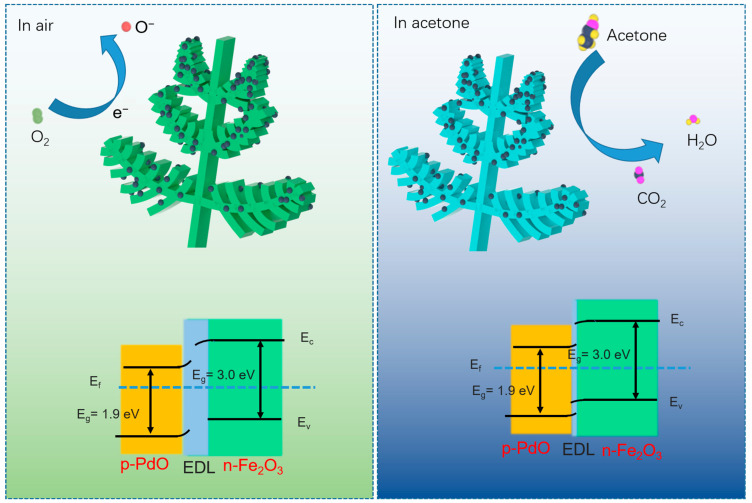
Schematic demonstration of the sensing mechanism of fern-like Pd/PdO-Fe_2_O_3_-based sensor toward acetone.

Differently from the pure Fe_2_O_3_ sensor, the sensing performance of the Pd/PdO-Fe_2_O_3_ sensor has been improved significantly. This result can be attributed to the following reasons. Firstly, the Pd/PdO noble metal on the surface of Fe_2_O_3_ accelerates the catalytic ionization of oxygen molecules to generate various oxygen species. In fact, noble metals have a high affinity for adsorbing gas molecules and can easily dissociate them into reactive atomic or ionic species. This dissociation lowers the activation energy required for the adsorbed molecules to interact with other surfaces. Once dissociated on the noble metal, these reactive species are inclined to migrate onto (spillover effect) the adjacent support material. In this case, oxygen atoms adsorbed on Pd/PdO can migrate to Fe_2_O_3_, promoting more effective electron exchange and enhancing sensor response. The adsorbed oxygen content is confirmed by XPS O1s characterization. Secondly, a Schottky barrier forms between grains due to surface depletion layers. The conductivity of the sensing material relies on electron transport across these grains. Electrons encounter high-energy barriers along their path, which hinder their movement and result in higher surface resistance in the sensor. As shown in Figure 5, a new heterojunction is formed at the interface of PdO and Fe_2_O_3_. The formation of a Fe_2_O_3_/PdO heterojunction is primarily driven by the work function difference between Fe_2_O_3_ and PdO. When these two materials come into contact, electrons will tend to flow from the material with the lower work function (Fe_2_O_3_) to the material with the higher work function (PdO). This electron transfer continues until an equilibrium is reached, resulting in the formation of a depletion layer at the Fe_2_O_3_/PdO interface. Finally, the porous and hierarchical nature of fern-like Fe_2_O_3_ promotes efficient gas diffusion throughout the structure. This allows for rapid interaction between gas molecules and the active sites, enhancing the response and recovery times of the sensor. Therefore, benefiting from the synergistic effect between Pd/PdO and Fe_2_O_3_, the sensor based on Pd/PdO-Fe_2_O_3_ shows excellent gas-sensing performance.

## 4. Conclusions

In summary, fern-like Pd/PdO-Fe_2_O_3_ with a hierarchical structure is successfully fabricated using a concise hydrothermal–reduction method to achieve enhanced gas-sensing performance. The gas-sensing properties of all Fe_2_O_3_-based samples are investigated comprehensively. It can be observed that the sensing properties of the fern-like Pd/PdO-Fe_2_O_3_ sensor are superior to that of the pure fern-like Fe_2_O_3_ sensor. Specifically, the response value of the fern-like Pd/PdO-Fe_2_O_3_ sensor to 100 ppm of acetone is about 73, exceeding those of its counterparts (47). In addition, the sensor based on fern-like Pd/PdO-Fe_2_O_3_ also shows excellent acetone selectivity and favorable long-term stability. This advancement of the fern-like Pd/PdO-Fe_2_O_3_ sensor in detecting acetone can be attributed to the synergistic effect between highly catalytically active Pd/PdO nanoparticles and fern-like Fe_2_O_3_. Thus, the sensor based on Pd/PdO-decorated fern-like Fe_2_O_3_ nanosheets is a promising candidate for high-performance acetone sensors.

## Data Availability

Data are contained within the article and Supplementary Materials.

## Supplementary Materials

The following supporting information can be downloaded at: https://www.mdpi.com/article/10.3390/molecules29235791/s1, Figure S1: Cross-section diagram of Pd/PdO-Fe_2_O_3_ sensor after sensing test at 180 °C; Figure S2: Humidity resistance capability of Pd/PdO-Fe_2_O_3_ sensor to 100 ppm of acetone at optimum working temperatures.
