# Supplementary material for: Bioinspired Fern-like Fe2O3 Functionalized with Pd/PdO Nanoparticles for High-Performance Acetone Sensing
Source: Molecules. 2024 Dec 7;29(23):5791. doi: 10.3390/molecules29235791 (PMC11643762; doi:10.3390/molecules29235791)
Supplement: Supplementary file 1 [file molecules-29-05791-s001.zip › molecules-3357816-supplementary.pdf]

Bioinspired Fern-like Fe_2O_3 Functionalized with Pd/PdO Nanoparticles for High-Performance Acetone Sensing

Gaohan Liu [1] and Haihang Wang [2,*]

[1] College of Materials Science and Engineering, Qingdao University, Qingdao 266071, China;
xiaonuanhan@163.com
[2] College of Materials Science and Engineering, Liaocheng University, Liaocheng 252000, China
* Correspondence: whh_qd@163.com

The sensing performance tests under varying target gas concentrations were conducted using the static liquid gas distribution method. The gas concentration was determined using the formula:

$$\mathrm{C(ppm)} = \frac{V1(mL) * \rho(g/mL) * 22.4\ (L/mol)}{M(g/mol) * V2\ (L)}$$

Here, C denotes the gas concentration, V1 and V2 are the volumes of the liquid and the test chamber, respectively, while ρ and M refer to the liquid's density and molecular weight.

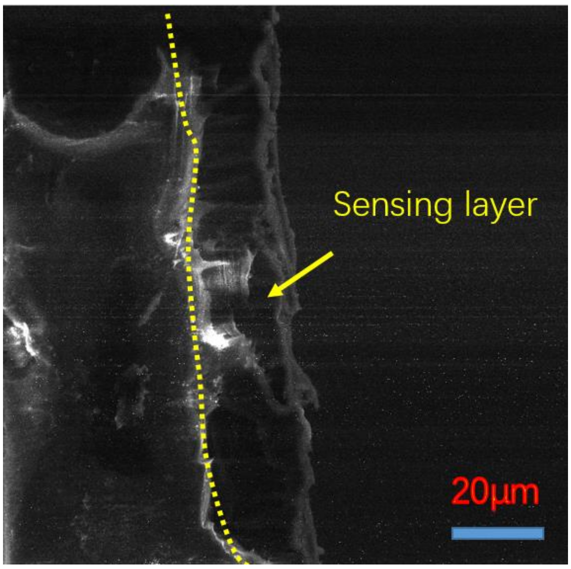

Figure S1. Cross-section diagram of Pd/PdO-Fe_2O_3 sensor after sensing test at 180°C.

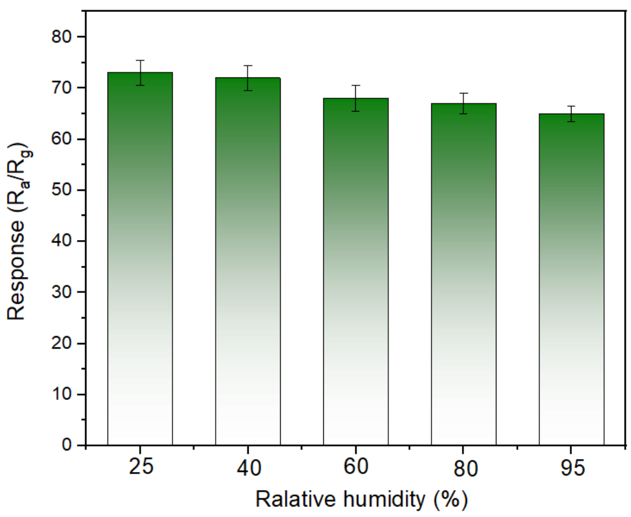

Figure S2. Humidity resistance capability of Pd/PdO-Fe_2O_3 sensor to 100 ppm of acetone at optimum working temperatures.
